# Trapezium fracture - a common technique to fix a rare injury: a case report

**DOI:** 10.4076/1757-1626-2-8304

**Published:** 2009-09-17

**Authors:** Darryl Nilesh Ramoutar, Constantine Katevu, Andrew Gordon Titchener, Arpit Patel

**Affiliations:** 1 Department of Trauma and Orthopaedics, Queens Medical Centre, Nottingham University Hospitals NHS TrustDerby Road, Nottingham, NG7 2UHUK; 2 Department of Trauma and Orthopaedics, Royal National Orthopaedic Hospital TrustBrockley Hill, Stanmore, Middlesex HA7 4LPUK; 3 Department of Trauma and Orthopaedics, Hinchingbrooke Hospital NHS TrustHinchingbrooke Park, Huntingdon, PE29 6NSUK

## Abstract

Trapezium fractures are rare injuries which should not be missed. We report a case of a 27-year-old right hand dominant man who sustained a closed vertical trapezium fracture and first carpometacarpal joint dislocation which was treated with closed reduction and percutaneous Kirschner-wire fixation, a technique familiar to all orthopaedic surgeons. Satisfactory functional outcome was achieved at final follow-up.

## Introduction

Isolated fracture of the trapezium is an uncommon injury accounting for only 3-5% of all carpal fractures [1]. However, they are very important fractures to detect and treat early given the importance of the trapezium in the carpometacarpal joint in actions such as grip and pinch. It is usually a result of a high energy trauma injury and can be classified into ridge and body fractures. Most are usually vertical body split fractures. Occasionally there may also be associated ligament damage (anterior oblique ligament, dorsoradial ligament, intermetacarpal ligament, posterior oblique ligament). Several methods have been described in the literature for treatment of this rare fracture, from conservative treatment in plaster to open reduction and internal fixation. We report a case of a fracture of the trapezium with associated dislocation of the first carpometacarpal joint which was treated with closed manipulation under anaesthesia and percutaneous Kirschner-wire insertion with good functional outcome at 6 months follow-up.

## Case presentation

**Figure 1.**
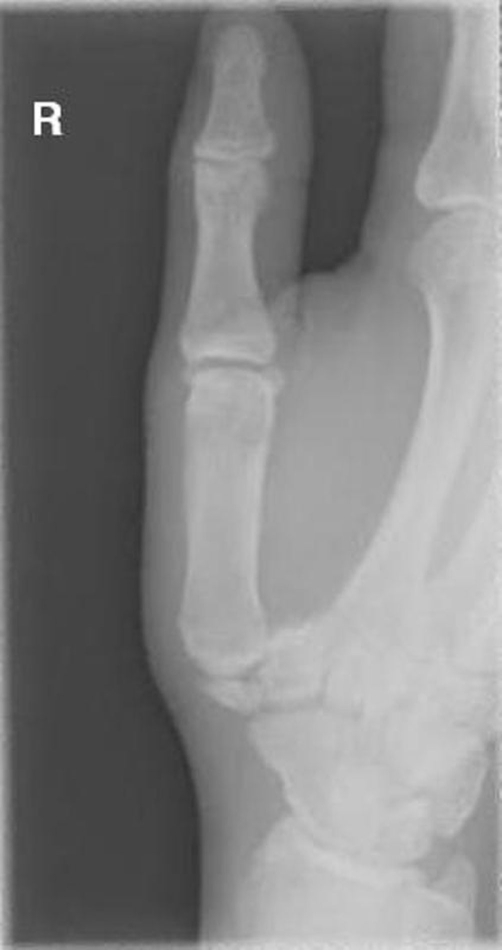
Plain radiograph of right hand post injury showing trapezium fracture and carpometacarpal dislocation.

**Figure 2.**
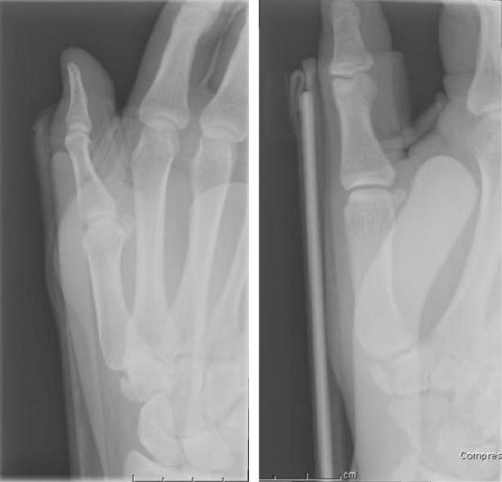
Plain radiograph of right hand post closed reduction of carpometacarpal dislocation.

**Figure 3.**
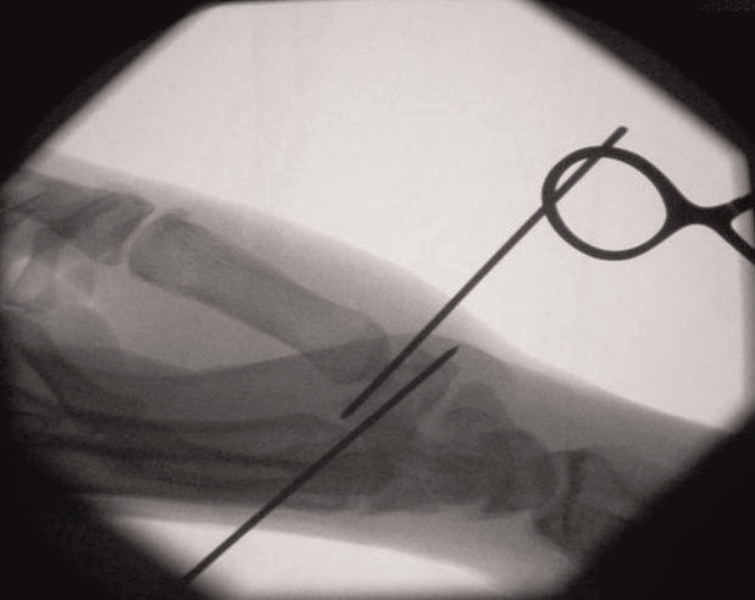
Image intensifier images of right hand post closed reduction and insertion of Kirschner wires.

**Figure 4.**
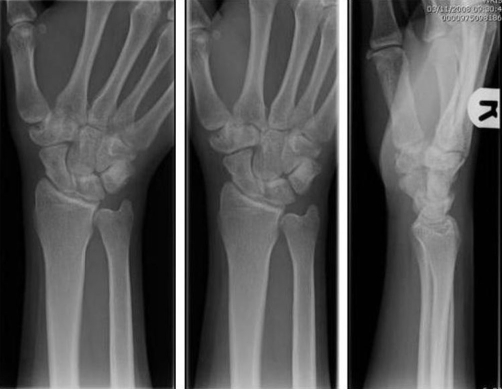
Check X-rays of right hand at 3 month follow-up.

A 27-year-old British Caucasian man presented to the Accident and Emergency department after falling on his extended right thumb while playing football. On clinical examination his right thumb was swollen and tender at the base of the first metacarpal with no tenderness in the anatomical snuff box. Movement of the thumb was limited secondary to pain and he was neurovascularly intact distally. Plain radiographs (Figure 1) revealed a vertical fracture of the trapezium with associated dislocation of the first carpometacarpal joint. This dislocation was reduced under analgesia and entonox in the department (Figure 2) and he was placed in a scaphoid plaster and referred to the fracture clinic. On review in the fracture clinic 2 days later it was decided that given the articular involvement associated with the injury, better anatomical reduction and stabilisation would be required. He was listed for surgical treatment which occurred 4 days post the initial date of his injury. He underwent closed manipulation of the fracture under anaesthesia until good position of the articular margin was achieved and this was fixed with 2 Kirschner-wires inserted under Image intensification via the edge of the anatomical snuff box to avoid injury to the radial artery. Check images were satisfactory (Figure 3). The joint was stable clinically and he was placed in plaster including the thumb. Check images at 8 weeks were satisfactory. The Kirschner-wires and plaster were removed and physiotherapy commenced. At 3 month (Figure 4) and 6 month follow-up the patient reported good functional outcome. Though we did not formally score his function, he reported satisfactory return to all his normal activities of daily living including writing, dressing and lifting. He had satisfactory grip strength and good range of movement of the thumb with minimal pain.

## Discussion

Trapezium fractures, with or without other associated injuries, are a rare but important diagnosis. The mechanism of injury usually involves either direct dorsoradial impaction or indirect axial loading. Though there are various types (ridge, body, vertical, transverse, comminuted), it is the indirect force mechanism that has been attributed to fractures of the trapezium body. The clinical presentation can be quite variable depending on the displacement of the fracture and the involvement of the carpometacarpal joint. Some patients only complain of minor pain at the base of the thumb without any gross swelling or deformity, whereas others, as in this case report, have swelling and severe restriction of movement. Thus, it is important to have a high clinical suspicion based on history and mechanism of injury. Imaging for this injury consists of plain radiographs, but often undisplaced fractures can be missed on these. A Robert’s AP view, with the hand in full pronation, is a good way of visualising the trapezium on plain radiographs. If the diagnosis is still in question Computerised Tomography or bone scintigraphy is recommended [2]. It is important to determine the stability of the joint before treatment. Especially in cases with associated dislocation, rupture of the surrounding ligaments and the dorsal joint capsule may result in instability even if the fracture itself is appropriately stabilised and these may require repair. Reconstruction of the inter-metacarpal and capsular structures, such as an inter-metacarpal abductor pollicis longus augmentation, as described by Brunelli et al [3] may be required, especially in isolated dislocations. This may not be necessary in fracture-subluxations, where the metacarpal base and dorsal trapezial fragment remain connected by the dorsal capsule. A temporary additional stabilising Kirschner wire will ensure inter-metacarpal orientation and relationship.

The literature reports several management options. However, as it is the universally accepted orthopaedic principle that fractures involving an articular surface require accurate reduction, most authors adhere to treatment involving accurate restoration of the articular surface. This is supported by two series [4,5] which highlighted the need for accurate reduction of the articular surface with displacement > 2mm. One article [6] reported successful conservative treatment of most undisplaced trapezium fractures in plaster cast only. However, another article [7] demonstrated dismal results in three patients with comminuted fractures treated this way.

Most of the literature recommends open reduction and internal fixation of vertically displaced intrarticular fractures of the trapezium as in this case. Corey and Ferrer-Torrells [8] were the first to recommend this and they used Kirschner wire-fixation for a series of five patients. Foster and Hastings [9] recommended either this or closed reduction and pinning, similar to what was done in this case. Inston et al [10] described the use of a Herbert screw which gave dynamic compression of the fragments and he reported very good success. Tolat and Jones [11] reported a case of a trapezium fracture with associated carpometacarpal dislocation in a skeletally immature 14 year old who was treated with accurate reduction and plaster and made a good recovery suggesting that accurate reduction/fixation of the trapezium may be enough to stabilise the trapezio-metacarpal joint. Recently, arthroscopic assisted fixation of this injury was reported. No studies have compared the outcome of Kirschner wire-fixation (percutaneous/open) to screw fixation (open) but Mcguigan and Culp [5] looked at 11 patients (largest series) with intra-articular trapezium fractures who had some type of surgical treatment and reported overall good outcomes. However it is important to note that 5/11 patients in this study showed degenerative changes at the trapeziometacarpal articulation at long-term follow-up (mean 47 months) despite excellent functional results at early review. Hence it is important to indicate this important long-term complication to all patients.

## Conclusion

We have reported a case of a trapezium fracture associated with carpometacarpal dislocation, a rare injury that was successful fixed using the relatively simple technique of closed reduction and percutaneous Kirschner-wire fixation with good functional outcome. This is a technique familiar to all orthopaedic surgeons and hence can be recommended as an alternative to using open reduction and screws in cases where satisfactory closed reduction can be achieved.
